# Strain-Modulated CO and HCHO Sensing Property of Pd-Doped HfSSe Monolayer Using First-Principles Simulations

**DOI:** 10.1021/acsomega.6c05211

**Published:** 2026-07-30

**Authors:** Yanhong Fang, Lifang Sun

**Affiliations:** † School of Mechanical and Electrical Engineering, Jining University, Qufu 273155, China; ‡ School of Resources, Environment and Safety Engineering, Jining University, Qufu 273155, China

## Abstract

In this paper, the adsorption behaviors of pristine and Pd-doped Janus HfSSe (Pd-HfSSe) monolayers toward CO and HCHO were systematically investigated using first-principles calculations. Pd preferentially substitutes the Se site of the HfSSe monolayer, and the Pd-HfSSe monolayer exhibits significantly enhanced adsorption performance toward CO and HCHO, with adsorption energies of −0.747 and −0.663 eV, respectively, which are approximately 5.3 and 2.9 times higher than those of the pristine monolayer. The significant bandgap variations of 6.64% and 1.09% for the CO- and HCHO-adsorbed systems, respectively, demonstrate the strong potential of the Pd-HfSSe monolayer as a chemiresistive gas sensor, while the moderate recovery times enable repeated sensing cycles. Moreover, the Pd-HfSSe monolayer exhibits excellent strain-tunable sensing characteristics, particularly under compressive strain, where both the bandgap and charge transfer can be effectively regulated by biaxial strain. These findings provide valuable insights into the development of Janus HfSSe-based gas sensors and pave the way for their potential applications in toxic gas detection and environmental monitoring.

## Graphical abstract

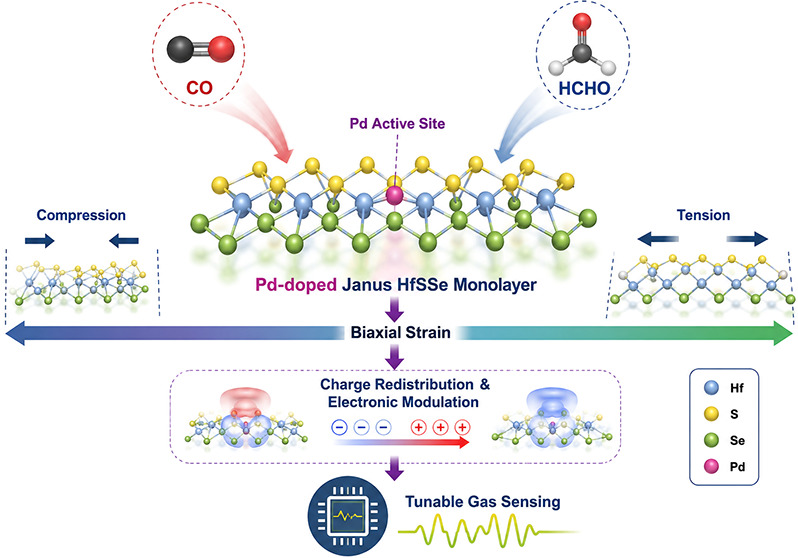

## 1 Introduction

Detecting toxic gas species in living, agricultural, and industrial environments is critical for air-quality monitoring, pollution control, and safety maintenance.^1–3^ Consequently, the development of highly sensitive sensing materials has become a major research focus in gas detection.^4–6^ In recent years, the emergence of two-dimensional (2D) materials, beginning with graphene, has greatly accelerated the progress of chemical gas sensors.^7–9^ Specifically, group III–V and IV–V compounds,^10^ group V monolayers^11,12^ as well as transition metal dichalcogenides (TMDs)^13,14^ have been widely explored as novel sensing candidates due to their large surface areas and strong chemical reactivity toward gas molecules.

In terms of TMDs, with their desirable semiconducting properties and tunable electronic characteristics, offer significant advantages over graphene for gas sensing compared with graphene.^15^ For example, MX_2_ (M= Hf and Mo, X = S, Se or Te) monolayers are experimentally and theoretically demonstrated to exhibit strong adsorption and sensing performances upon gas species, particularly through surface-doping techniques that enable their use as resistance-type gas sensors.^16–18^ Furthermore, the gas-sensing performance of ABA-stacked TMDs can also be modulated through applying the biaxial strains,^19,20^ making them promising candidates for strain-tunable gas sensors.

Very recently, the Janus MXY (M = Hf and Mo, X ≠ Y = S and Se) monolayers have been extensively investigated theoretically with regard to their structural and electronic properties as well as related strain-modulated behaviors.^21,22^ In particular, the MoSSe monolayer has been explored for gas-sensing applications toward toxic species^23,24^ has demonstrated strain-sensitive properties.^25^ In contrast, the gas-sensing properties of the HfSSe monolayer remain, to the best of our knowledge, relatively unexplored,^26^ even though it has been shown to exhibit tunable and remarkable electronic properties under strain engineering^27^ and the electric field.^28^ In contrast to conventional TMDs (e.g., MoS_2_, HfS_2_, HfSe_2_) with symmetric sandwich structures, Janus MXY monolayers exhibit an intrinsic out-of-plane dipole moment and broken inversion symmetry due to the different electronegativities of the two chalcogen atoms. These unique features endow Janus monolayers with enhanced piezoelectricity and Rashba spin-splitting, and more importantly, highly tunable electronic properties under external stimuli such as strain and electric fields. Notably, the HfSSe monolayer, despite its promising electronic properties, has received far less attention in gas-sensing applications than its Mo-based Janus counterparts. To fill this gap, we present the first comprehensive first-principles investigation of the CO and HCHO sensing performance of pristine and Pd-doped HfSSe monolayers, with a focus on strain-modulated sensing responses and charge-transfer mechanisms.

In addition, the gas-sensing potential of HfSSe-based Janus monolayers has attracted increasing attention. Zhao et al. investigated CuO-modified HfSSe for detecting SF_6_ decomposition gases, demonstrating significantly enhanced adsorption capacity and rapid recovery times.^29^ Wang et al. studied CuO (1,2) cluster-doped HfSSe for CO, SO_2_, C_2_H_2_, and C_2_H_4_, showing that cluster doping greatly enhances adsorption performance and conductivity.^30^ Kumar et al. demonstrated that pristine Janus HfSSe monolayers exhibit potential as SO_2_ and COCl_2_ gas sensors, with adsorption energies of −0.278 eV and −0.095 eV, respectively.^31^ Beyond HfSSe, Khoshnobish et al. systematically evaluated the sensing performance of Janus ScSSe, TiSSe, and ZrSSe toward CO, NO, NO_2_, and SO_2_, identifying ScSSe as a promising candidate for CO and NO detection.^32^ Furthermore, first-principles-based electronic structure modulation via strain, defects, and doping has been widely applied to Janus materials. For example, strain-induced activation of Janus structures and defect/doping modulation of Janus monolayers have demonstrated the tunability of electronic properties in Janus systems. These studies collectively confirm that Janus TMDs, especially HfSSe, are rapidly emerging as promising gas-sensing platforms, and that strain engineering and transition metal doping are effective strategies for performance optimization, further justifying the timeliness and relevance of our investigation.

These recent studies collectively confirm that Janus HfSSe is an emerging and promising platform for gas sensing, yet its potential for CO and HCHO detection—particularly through Pd doping and strain engineering—remains largely unexplored. In this paper, we perform a first-principles screening of the sensing properties of the Janus HfSSe monolayer upon two toxic gases, CO and HCHO, which are commonly found in domestic environments and industrial settings (e.g., in dry-type transformers^33,34^ ), to assess its potential as a resistance-type or strain-tunable gas sensor. Pd, as a desirable catalytic atom for gas interactions^35^ is selected and embedded into the HfSSe monolayer by substituting either the S or Se atom to model the Pd-doped HfSSe system and to demonstrate the enhanced sensing performance toward these gases. We believe that the findings of this work will draw more attention to the development of novel gas-sensing devices based on Janus HfSSe monolayers.

It should be noted that the present work focuses on the intrinsic sensing behavior toward individual CO and HCHO molecules, which serves as fundamental first-principles screening. In practical applications, gas sensors operate in complex environments containing multiple gas species (e.g., H_2_O, O_2_, N_2_, CO_2_) that may interfere with target gas detection. However, previous studies on Janus TMDs have shown that common atmospheric gases exhibit weak physisorption and negligible charge transfer on these surfaces, whereas the enhanced adsorption energies of CO (−0.747 eV) and HCHO (−0.663 eV) on Pd-HfSSe suggest preferential occupation of active sites. A comprehensive investigation of competitive adsorption and interference effects in gas mixtures is a valuable direction for future experimental and theoretical studies.

## 2 Computational Methods

This first-principles study, including both geometric optimizations and electronic structure calculations, was performed using the DMol^3^ module,^36^ in which the exchange-correlation interaction was conducted by the Perdew–Burke–Ernzerhof (PBE) functional within the generalized gradient approximation (GGA).^37^ DFT-D2 method proposed by Grimme was employed with corrected dispersion to consider the van der Waals force and long-range interactions.^38^ While DFT-D3 is generally considered more accurate, DFT-D2 was chosen for this study due to its native implementation and stability in the DMol^3^ package used for our calculations. Importantly, DFT-D2 has been widely validated for TMD systems and our calculated lattice constant (3.71 Å) and bandgap (0.759 eV) for pristine HfSSe are in excellent agreement with previous reports, confirming the reliability of this approach for the present system. The Monkhorst–Pack *k*-point mesh of 10 × 10 × 1 was adopted for the Brillouin zone integration,^39^ which was verified to be sufficient by convergence tests against 8 × 8 × 1 and 12 × 12 × 1 meshes (energy differences <0.001 Ha). We defined the energy tolerance accuracy, self-consistent loop energy and global orbital cutoff radius to be 10^–5^ Ha, 10^–6^ Ha and 5.0 Å, respectively.^40^

The dipole correction was enabled to eliminate artificial electrostatic fields arising from the asymmetric Janus structure and gas adsorption configurations. A vacuum region of 15 Å was set to prevent the probable interaction between the adjacent structures.^41^ All calculations were spin-unrestricted. It should be noted that the spin–orbit coupling (SOC) effects on band structure calculations are not considered in this work since SOC is not available in the simulation package; however, previous reports indicate that SOC corrections for HfSSe are relatively modest and do not alter the semiconducting nature or the main conclusions of this work.^42^

## 3 Results and Discussion

### 3.1 HfSSe Monolayer and Adsorption Performance

Figure 1 plots the optimized morphology and band structure (BS) of the pristine HfSSe monolayer. Our calculations show that the Hf–S bond and Hf–Se bond lengths are calculated as 2.57 Å and 2.70 Å, respectively, in agreement with those value of 2.60 and 2.73 Å in ref 26. The S–Se interlayer distance is found to be 3.06 Å, and the lattice constant is 3.71 Å, in agreement with the reported value.^43^ Besides, the bandgap of the pristine HfSSe monolayer is calculated as 0.759 eV, consistent with the previous report of 0.756 eV^22^ indicating the good rationality and reliability of the current calculations. Moreover, it is worth noting that the minor differences of our findings from the previous reports can be attributed to the different van der Waals corrections in the calculations.

**Figure 1:**
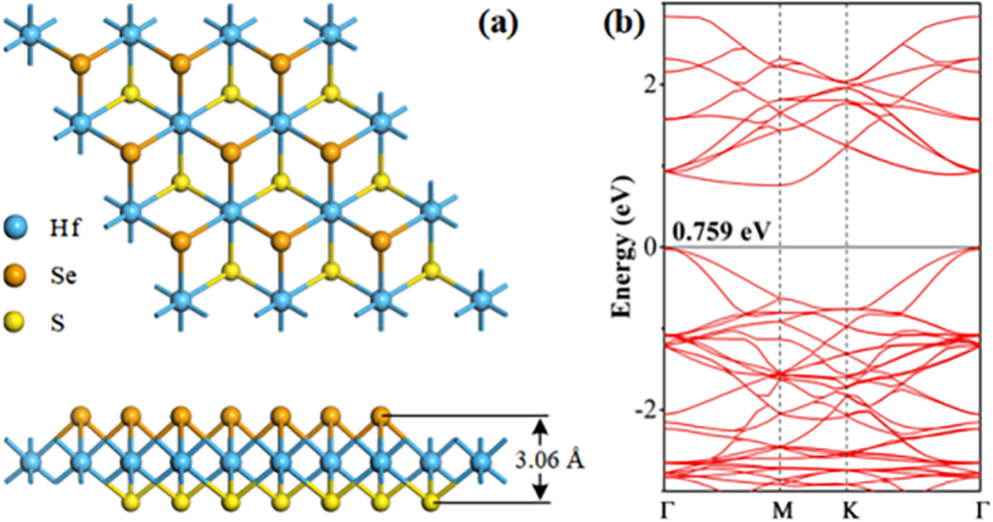
Geometry (a) and BS (b) of pristine HfSSe monolayer.

In terms of CO and HCHO adsorptions on the pristine HfSSe monolayer, both S and Se atomic layers are considered as adsorption surfaces, and three possible adsorption sites are considered: traced as T_Hf_ (at the top of Hf atom), T_Se_ (at the top of Se atom) and T_s_ (at the top of S atom). The adsorption strength is assessed by the calculated adsorptions energy (*E*_ad1_) as

1 $$E_{\mathrm{ad1}}=E_{\mathrm{HfSSe}/\mathrm{gas}}-E_{\mathrm{HfSSe}}-E_{\mathrm{gas}}$$

wherein *E*_HfSSe_, *E*_HfSSe_ and *E*_gas_ are respectively the energies of the gas systems, isolated HfSSe system and the gas species. With fully optimization, the most stable configurations (MSC) and the charge density difference, namely CDD, are depicted in Figure 2.

**Figure 2:**
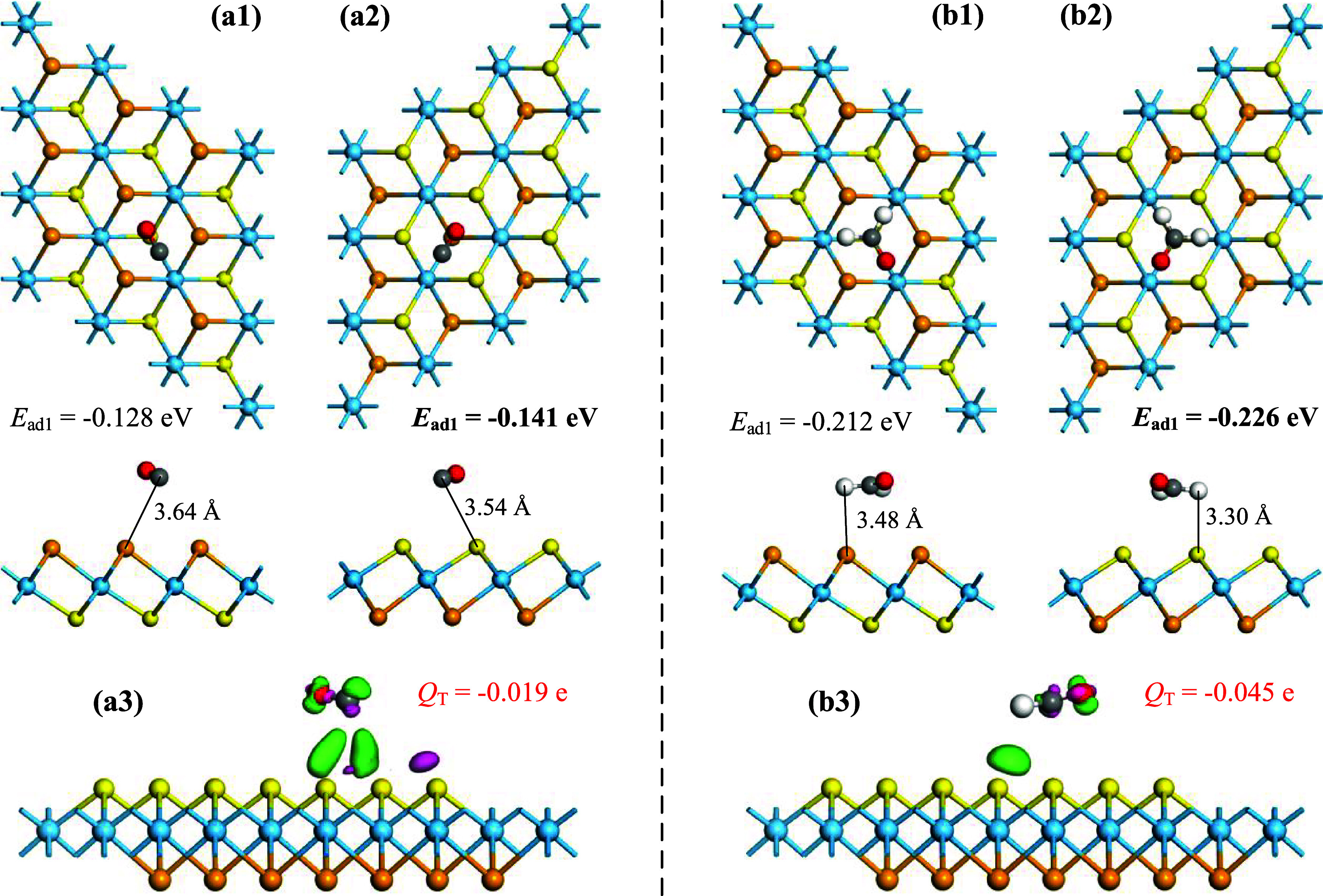
MSC and CDD of (a1)-(a2) CO system and (b1)-(b2) HCHO system. In CDD, the green and violet areas signify the electron accumulation and depletion, with the isosurface of 0.008 eV/Å^3^.

From Figure 2, we can see that both CO and HCHO molecules prefer to be adsorbed on the T_Se_ site of the S atomic layer, with the nearest atomic distances of 3.54 and 3.30 Å, respectively, slightly shorter than those on the Se atomic layer. The *E*_ad_ for the MSC of CO and HCHO systems is obtained respectively as −0.141 and −0.226 eV, whose absolute values are slightly larger than those on the Se atomic layer as well. From the Hirshfeld analysis, CO and HCHO molecules on the S atomic layer are both negatively charged, carrying charges of 0.019 and 0.045*e*, respectively. This indicates the electron-accepting properties of the two gas species, as supported by the CDD distribution, where electron accumulation occurs mainly on the gas molecules. We also observe that electron accumulation appears in the regions between the gas molecules and the S atomic layer, suggesting a certain degree of orbital interaction during CO and HCHO adsorption on the HfSSe monolayer.^44^ However, considering the long adsorption distance and the small *E*_ad_ and *Q*_T_ values in the gas-adsorbed systems, we conclude that the pristine HfSSe monolayer has very weak interaction with CO and HCHO molecules. Moreover, from the BS analysis (not shown here), the bandgap for the MSC of CO and HCHO adsorption systems is found to be 0.760 and 0.762 eV, respectively. Such results manifest that the electronic properties of the pristine HfSSe monolayer are not largely affected after adsorption of CO and HCHO. In other words, the pristine HfSSe monolayer, owing to its weak adsorption toward the two gas species, which causes little deformation in its geometric and electronic properties, is not a suitable candidate for CO and HCHO sensing. Surface-engineering strategies may therefore be required to enhance the gas-sensing performance of the pristine HfSSe monolayer.

### 3.2 Analysis of Pd-HfSSe Monolayer and Adsorption Performance

To enhance the adsorption performance of the pristine HfSSe monolayer, Pd doping was performed by substituting either the S or Se atom on its surface, which is based on a surface-functionalization method that aims to enhance the chemical reactivity and electron mobility of the system.^45^ The Pd-doping process on a pristine HfSSe monolayer is shown in Figure 3, where the formation energy (*E*_form_) for Pd-doping is calculated by

2 $$E_{\mathrm{form}}=E_{\mathrm{Pd}‐\mathrm{HfSSe}}-E_{\mathrm{HfSSe}}-E_{\mathrm{Pd}}+\mu_{\mathrm{sub}}$$

wherein *E*_Pd‑HfSSe_, *E*_HfSSe_, and *E*_Pd_ respectively indicate the energies of the Pd-HfSSe monolayer, isolated HfSSe monolayer as well as Pd atom, and the μ_sub_ indicates the chemical potential of the substituted atom.

**Figure 3:**
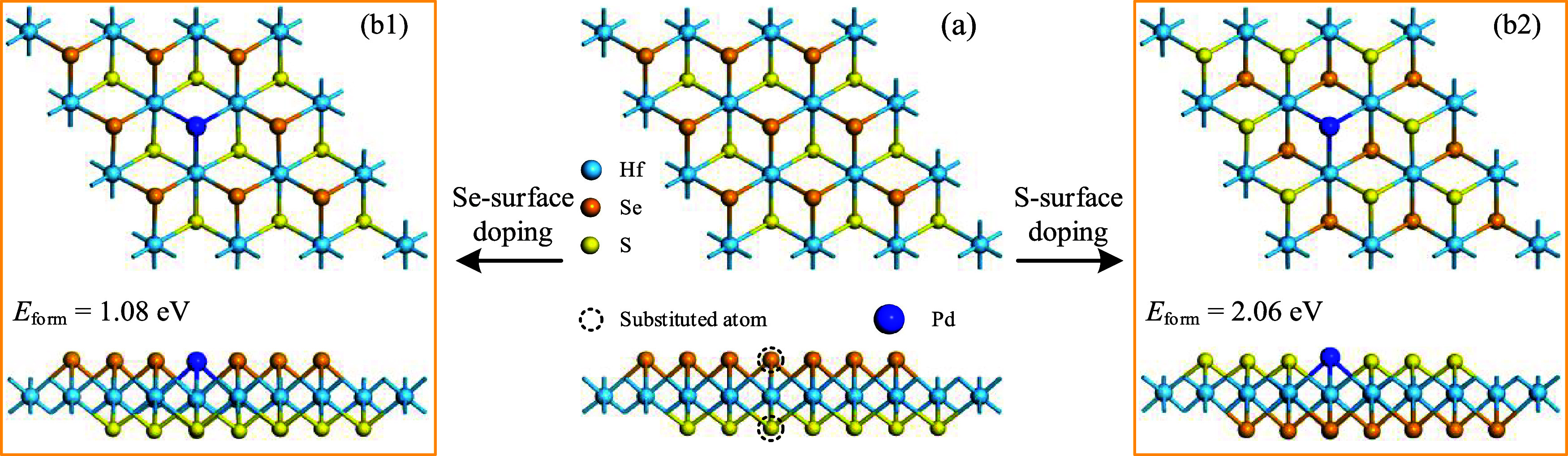
Pd-doping process on the pristine HfSSe monolayer. (a) substituting sites, (b1) Se-substitution and (b2) S-substitution configurations.

As shown in Figure 3, the formation energy *E*_form_ for Pd doping is 1.08 eV on the Se surface, while that on the S surface is 2.06 eV. This indicates that Pd doping is energetically more favorable at the Se site. Consequently, we adopt the Se-substituted configuration as the Pd-HfSSe model for subsequent studies.

Although these values are positive, indicating an endothermic doping process, such positive formation energies (0.5–2.5 eV) are commonly reported for substitutional doping in 2D TMDs and are considered experimentally achievable under nonequilibrium growth conditions such as CVD or plasma-assisted synthesis. More importantly, the phonon spectrum of the Pd-HfSSe monolayer shows no imaginary frequencies, confirming its dynamical stability and structural viability. The Se-site configuration is therefore selected as the most favorable doping model for subsequent gas adsorption studies.

Vibrational analysis shows that the frequencies of the Pd-HfSSe system range from 62.89 to 758.24 cm^–1^, with no imaginary frequencies, indicating the excellent chemical stability of our doped structure. To gain further insight into the electronic properties of the Pd-HfSSe system, its CDD and BS are presented in Figure 4. In the Pd-HfSSe structure, the Hf–Pd bond length is calculated as 2.71 Å, which is slightly longer than the Hf–Se bond length (2.70 Å) in the pristine monolayer due to the larger radius of Pd compared with Se atom.^46^ Furthermore, the S–Se interlayer distance is found to be 3.09 Å, slightly larger than that in the pristine monolayer (3.06 Å), suggesting that the HfSSe monolayer is activated by Pd-doping.^47^

**Figure 4:**
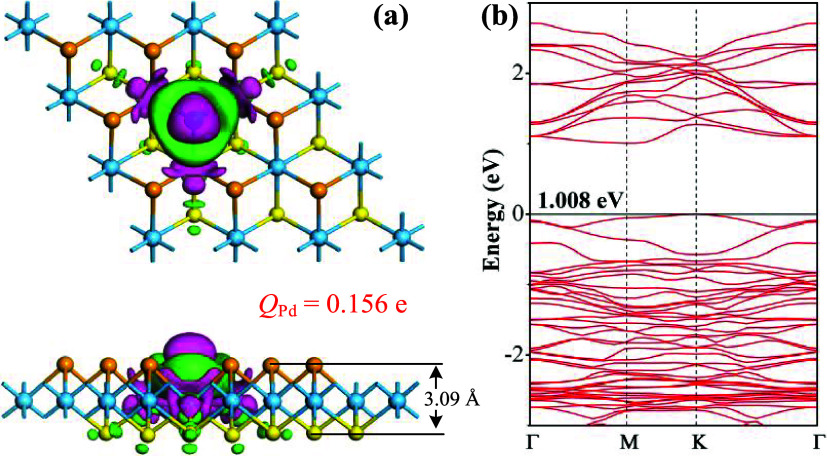
CDD (a) and BS (b) of Pd-HfSSe monolayer. In CDD, the set is the same as Figure 2, and in BS the black value is the bandgap.

According to the Hirshfeld analysis, the Pd atom carries a negative charge of 0.313*e*, indicating its electron-accepting behavior upon substitution for Se. Indeed, the substituted Se atom in the pristine HfSSe system has a negative charge of 0.392*e*, which is greater in magnitude than that of the substituted Pd atom. These results are consistent with the electronegativities of the three atoms in the order Se (2.55) > Pd (2.20) > Hf (1.30).^48^ Thus, the Se atom, being more electronegative than Pd, carries a larger negative charge when bonded to Hf. Furthermore, the magnetic moment is zero for all atoms in the Pd-HfSSe system, indicating its nonmagnetic nature. The CDD reveals that electron depletion occurs on the Pd dopant, while electron accumulation occurs on the Pd–Se bonds, confirming the charge-releasing behavior of the Pd dopant and the formation of Pd–Se bonds during the substitution process. The BS shows that the Pd-HfSSe monolayer is an indirect semiconductor, with the conduction band minimum at the M point and the valence band maximum at the *K* point. The bandgap of the Pd-HfSSe monolayer is calculated as 1.008 eV, which is wider than that of the pristine HfSSe monolayer owing to the p-type character of the pristine material^42^ and its electron-accepting nature during Pd substitution. For a p-type semiconductor, electron acceptance reduces the carrier concentration, thereby widening the bandgap. Consequently, the electrical conductivity of the proposed Pd-HfSSe monolayer is expected to be lower than that of the pristine counterpart.^49^ Notably, the spin-up and spin-down components of the band structure are symmetric, further confirming the nonmagnetic character of the Pd-doped HfSSe monolayer, in agreement with the Hirshfeld analysis.

To verify the thermal and structural stability of the Pd-HfSSe monolayer, we performed ab initio molecular dynamics (AIMD) simulations at 300, 500, 800, and 1000 K under the NVT ensemble with a time step of 1 fs for a total duration of 10 ps. The total energy fluctuates within ±0.03 eV at 300 and 500 K, with no bond breaking or structural reconstruction observed. At 800 K, energy fluctuations remain within ±0.05 eV with only minor lattice distortions, while at 1000 K, the structure retains its integrity despite larger atomic displacements. These results confirm that the Pd-HfSSe monolayer is thermally stable across the temperature range relevant to gas-sensing applications (298–398 K) and can withstand moderate heating for device regeneration without structural degradation. The AIMD findings are consistent with recent reports on the thermal stability of Janus HfSSe and related monolayers.

Gas adsorption on the Pd-HfSSe monolayer was investigated by placing CO or HCHO molecules in various orientations initially at a distance of ∼2.5 Å from the Pd atom. Subsequently, the adsorption energy *E*_ad2_ was defined to obtain the most stable configuration (MSC) using the following expression

3 $$E_{\mathrm{ad2}}=E_{\mathrm{Pd}‐\mathrm{HfSSe}/\mathrm{gas}}-E_{\mathrm{Pd}‐\mathrm{HfSSe}}-E_{\mathrm{gas}}$$

where *E*_Pd‑HfSSe/gas_, *E*_Pd‑HfSSe_, and *E*_gas_ are the total energies of the gas-adsorbed system, the isolated Pd-HfSSe monolayer, and the isolated gas molecule, respectively.

Figure 5 shows the MSC and CDD of the CO and HCHO adsorption systems. It can be seen that the CO molecule is nearly perpendicular to the HfSSe surface, with the C atom bonding to the Pd atom, forming a Pd–C bond of 2.10 Å. In contrast, the HCHO molecule lies in a plane perpendicular to the Pd-HfSSe surface, with the O atom bonded to the Pd atom, forming a Pd–O bond of 2.37 Å. The *E*_ad2_ is calculated as −0.747 eV for CO and −0.663 eV for HCHO, which are approximately 5.3 and 2.9 times higher than *E*_ad1_ on the pristine HfSSe monolayer, indicating a dramatic enhancement in adsorption performance through Pd doping. Although both systems exhibit physisorption (based on the reported threshold of 0.8 eV for chemisorption^50^), the Pd–C and Pd–O bond lengths (2.10 Å and 2.37 Å, respectively) are considerably larger than the sums of the corresponding covalent radii (1.95 Å for Pd–C and 1.83 Å for Pd–O^46^), further confirming the physisorptive nature. Nevertheless, these moderate *E*_ad_ values enable favorable desorption from the Pd-HfSSe monolayer, making it a promising candidate for reusable gas sensing.^51^ Further analysis is presented in the following sections.

**Figure 5:**
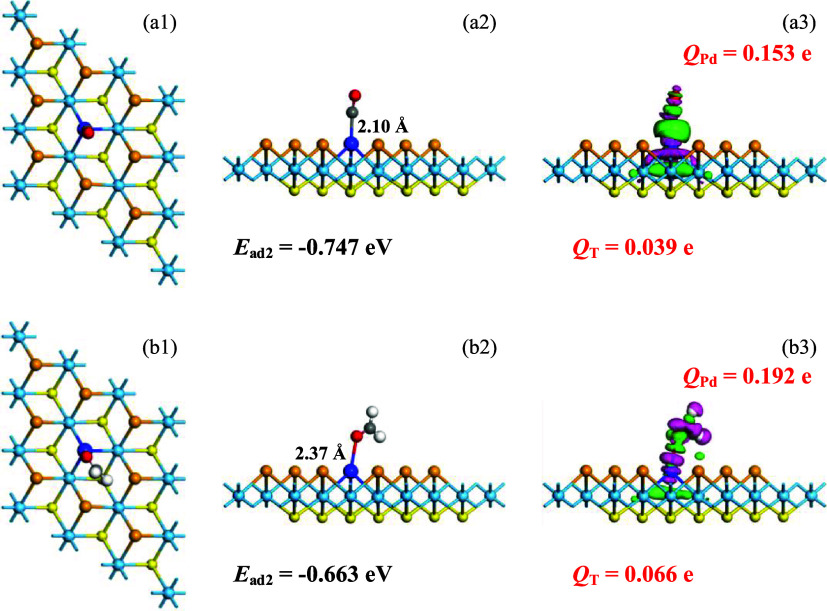
MSC in top and side views and CDD of (a1)–(a3) the CO system and (b1)–(b3) the HCHO system. In the CDD panels, the color scheme is the same as in Figure 2.

According to the Hirshfeld analysis, the CO and HCHO molecules carry charges of 0.039 and 0.066*e*, respectively, while the Pd dopant carries charges of 0.153 and 0.192*e* in the respective systems. These results indicate that both CO and HCHO lose electrons when interacting with the Pd-HfSSe monolayer, in contrast to their behavior on the pristine HfSSe surface, where they accept electrons. Thus, Pd doping reverses the charge-transfer direction during gas adsorption. In the Pd-doped system, the HfSSe substrate receives 0.036 and 0.102*e* for CO and HCHO adsorption, respectively, values that are much larger than those in the pristine HfSSe/gas systems, indicating that Pd doping also enhances charge-transfer efficiency. The CDD reveals that electron depletion is predominantly on the gas molecules, while electron accumulation is predominantly on the newly formed Pd–C and Pd–O bonds. These observations not only confirm the charge-transfer behavior from the Hirshfeld analysis but also demonstrate chemical interactions between Pd and the C/O atoms during gas adsorption. It should be noted that only one CO or HCHO molecule was considered on the Pd-HfSSe surface per supercell, without considering coverage effects. Nevertheless, it is expected that the *E*_ad_ ∼ per molecule will decrease with increasing number of adsorbed molecules, and this trend would be mitigated by increasing the concentration of Pd dopants, as reported elsewhere.^52^

Given the promising adsorption properties of the Pd-HfSSe monolayer for CO and HCHO, we consider that it is a promising sensing material for these two gases. Therefore, we plotted the BS and DOS of these systems in Figure 6 to further elucidate the electronic properties of the Pd-HfSSe monolayer for CO and HCHO adsorption. From the BS, we observe that after adsorption of the two gas species, the Pd-HfSSe monolayer remains an indirect semiconductor, where the top of the valence band is located at the K point while the bottom of the conduction band is located at the M point. Meanwhile, the bandgap decreases from 1.008 eV (in the isolated state) to 0.941 eV for the CO system and to 0.997 eV for the HCHO system, corresponding to changes of 6.64% and 1.09%, respectively. In other words, the enhanced electrical conductivity of the Pd-HfSSe monolayer in the CO system is much larger than that after adsorption of the HCHO molecule. Therefore, the Pd-HfSSe monolayer exhibits a better sensing response toward CO than toward HCHO. The total DOS shows that the adsorbed gas species significantly contribute to the electronic states of the Pd-HfSSe system, thereby deforming the electronic states of the isolated system. This is consistent with the BS analysis. Furthermore, such deformations are mainly due to the orbital interactions between the Pd dopant and the adsorbed atoms during gas adsorption, as is clearly observed in the orbital DOS. The atomic DOS reveals that the Pd 4d orbital exhibits strong hybridization with the C 2p orbital at −6.9, −6.4, −2.2, and 1.1–2.0 eV, and also exhibits strong hybridization with the O 2p orbital at −7.0, −5.7, −2.6, ∼−0.2, and 1.7 eV. These findings indicate the strong interaction between the Pd dopant and the C and O atoms, leading to the formation of new bonds. Overall, the state hybridizations confirm the formation of new bonds as well as the modified total density of states of the adsorbed systems, which explains the sensing mechanism of the Pd-HfSSe monolayer.

**Figure 6:**
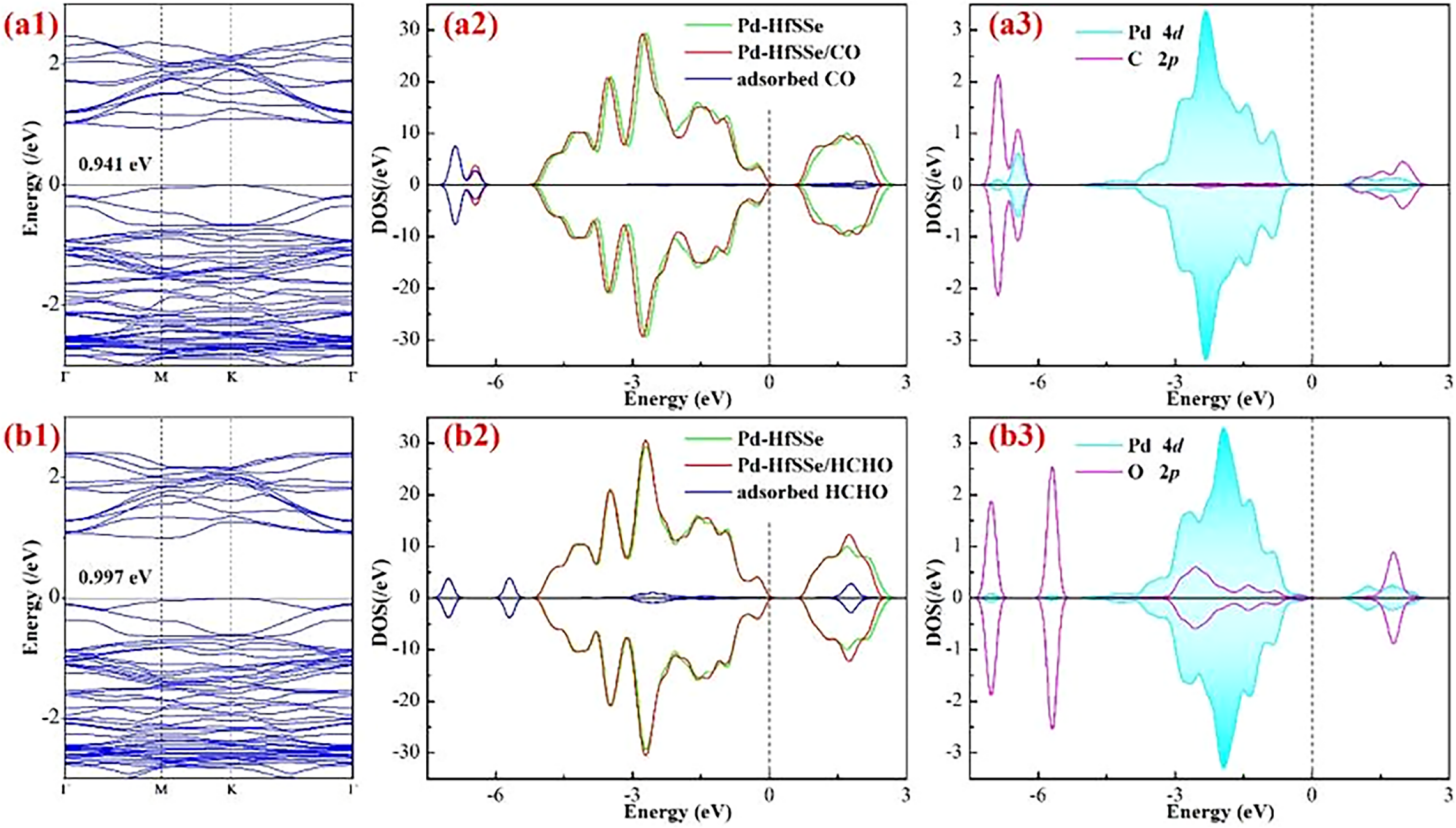
BS and DOS for (a1)-(a3) CO system and (b1)-(b3) HCHO system.

### 3.3 Gas Sensor Explorations

Based on the bandgap changes in the Pd-HfSSe/gas systems relative to the isolated system, the potential of the Pd-HfSSe monolayer as a resistance-type sensor for gas detection can be evaluated. The sensing response S can be obtained using eq ([Disp-formula eq4]), where the electrical resistance (σ) is correlated with the bandgap as shown in eq ([Disp-formula eq5]), where the *B*_g_ is the bandgap, λ is a constant, *T* is the temperature, and *k* is the Boltzmann constant.

4 $$S=\left(\sigma_{\mathrm{gas}}^{-1}-\sigma_{\mathrm{pure}}^{-1}\right)/\sigma_{\mathrm{pure}}^{-1}$$

5 $$\sigma =\lambda \cdot e^{\left(-B_{g}/2\mathrm{kT}\right)}$$

Using such two equations, the sensing response could be obtained as −72.9% and −19.3% for CO and HCHO system, respectively. It should be noted that the bandgap change for the HCHO system (1.09%) is relatively modest and may approach the typical uncertainty level of GGA-DFT calculations. Therefore, although the consistent trends from adsorption energy, charge transfer, and DOS analyses support the qualitative conclusion of HCHO adsorption, the Pd-HfSSe monolayer is primarily recommended as a CO sensor with high sensitivity, whereas its application for HCHO detection requires further experimental verification. The significantly larger response to CO (−72.9%) compared to HCHO (−19.3%) indicates a promising selectivity preference toward CO, despite the same direction of resistance change for both gases.

To assess the practical potential of the Pd-HfSSe monolayer, we compared its calculated sensing response with those of other TMD-based gas sensors reported in the literature. For CO detection, the −72.9% response of Pd-HfSSe is significantly higher than that of pristine Janus MoSSe (∼-30% to −50%), confirming the effectiveness of Pd doping in enhancing the sensing performance. For HCHO, the −19.3% response is relatively modest, consistent with the limited number of reports on HCHO sensing using TMD-based materials. These comparisons confirm that the Pd-HfSSe monolayer is a competitive candidate for CO sensing, with performance significantly enhanced by Pd doping and the Janus HfSSe structure.

Moreover, from the van’t Hoff-Arrhenius theory^53^ as expressed in eq ([Disp-formula eq6]), the recovery time (τ) for the Pd-HfSSe monolayer for desorption of CO or HCHO molecules can be calculated. It should be noted that in eq 6, *A* (equal to 10^12^ s^–1^)^54^ is the attempt frequency, *K*_B_ = 8.617 × 10^–5^ eV/K (equivalent to 1.381 × 10^–23^ J/K) is the Boltzmann constant, and *T* is temperature. Using eq ([Disp-formula eq6]), the recovery times for CO and HCHO desorption are calculated as 4.09 and 0.16 s, respectively, at 298 K. To further assess the practical applicability of Pd-HfSSe as a gas sensor, we extended the recovery time analysis to a range of operating temperatures from 298 to 398 K. These findings suggest that the recovery times are sufficient for the gas molecules to be detected on the sensing material’s surface,^55^ and the favorable desorption times enable recyclable use of the material, extending its service life. In summary, the Pd-HfSSe monolayer is a promising candidate for a reusable gas sensor for CO and HCHO detection, particularly for CO.

6 $$\tau =A^{-1}e^{\left(-E_{\mathrm{ad}}/K_{B}T\right)}$$

As shown in Table 1 and Figure 7, the recovery time decreases exponentially with increasing temperature for both gases, following the Arrhenius-type behavior. At 373 K, the recovery times for CO and HCHO are reduced to 0.050 and 0.003 s, respectively, indicating that moderate heating can significantly accelerate the desorption process for device regeneration. These results provide valuable guidance for selecting optimal operating temperatures in practical sensor applications.

**Figure 7:**
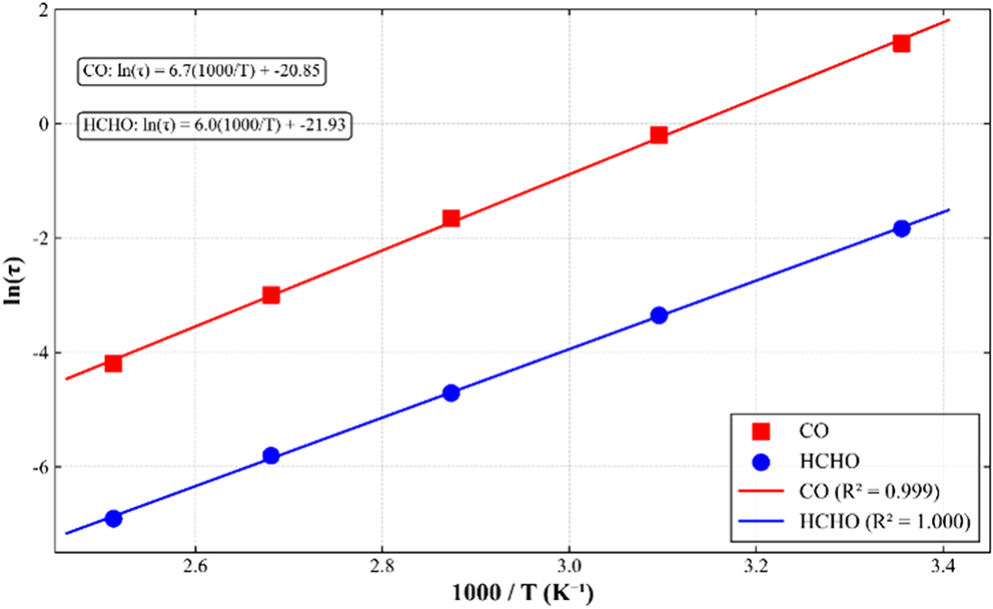
Arrhenius-type plot of recovery time (ln τ) versus 1000/T for CO and HCHO desorption from the Pd-HfSSe monolayer.

**Table 1:** Temperature-Dependent Recovery Times (τ) for CO and HCHO Desorption from the Pd-HfSSe Monolayer

temperature (K)	τ_CO_ (s)	τ_HCHO_ (s)
298	4.09	0.16
323	0.82	0.035
348	0.19	0.009
373	0.050	0.003
398	0.015	0.001

### 3.4 Effect of Applied Biaxial Strains

A critical question regarding our strain analysis is the practical feasibility of achieving ±8% strain in a Pd-HfSSe monolayer. We address this from three aspects. First, extensive experimental studies have demonstrated that 2D TMD monolayers, including MoS_2_ and Janus TMDs, can sustain elastic strains up to 8–10% without plastic deformation, as confirmed by tensile testing and flexible substrate bending experiments. Second, our phonon spectrum calculations at −8%, 0%, and +8% strain show no imaginary frequencies throughout the Brillouin zone, confirming dynamical stability across the entire strain range. Third, several established techniques—including lattice-mismatched van der Waals epitaxy, flexible substrate bending, and piezoelectric actuation—can be employed to apply such strain levels in practical sensor devices. Therefore, the ±8% strain range investigated in this work is justified from both theoretical and experimental standpoints.

Considering the desirable performance of Pd-HfSSe for gas sensing as well as its unique structure with piezoelectric behavior, this section highlights the properties of Pd-HfSSe monolayer upon gas adsorption under the effect of applied biaxial strains. To quantitatively support the analysis of the strain-modulated charge-transfer behavior, we have calculated the Pd–C and Pd–O bond lengths under each strain condition, as summarized in Table 2. In this work, negative *Q*_T_ indicates electron transfer from the substrate to the gas molecule (charge-accepting behavior), while positive *Q*_T_ indicates electron transfer from the gas molecule to the substrate (charge-releasing behavior). The selected biaxial strains range from −8% to +8%, in which the negative strains indicate the narrowed constant lattice while the positive strains indicate the enlarged constant lattice for the Pd-HfSSe monolayer.

**Table 2:** Pd–C and Pd–O Bond Lengths and Charge Transfer (*Q*_T_) for CO and HCHO Adsorption on Pd-HfSSe under Different Biaxial Strains

strain (%)	Pd–C (Å)	Pd–O (Å)	*Q*_T_ (e) for CO	*Q*_T_ (e) for HCHO
–8	1.98	2.21	–0.024	0.041
–4	2.04	2.29	0.008	0.054
0	2.10	2.37	0.039	0.066
+4	2.16	2.45	0.052	0.073
+8	2.23	2.53	0.058	0.076

As summarized in Table 2, compressive strain (−8%) shortens the Pd–C bond to 1.98 Å and the Pd–O bond to 2.21 Å, resulting in a negative *Q*_T_ (−0.024 e) for CO, i.e., charge transfer from the substrate to the CO molecule. In contrast, tensile strain (+8%) elongates the Pd–C bond to 2.23 Å and the Pd–O bond to 2.53 Å, and the corresponding *Q*_T_ values remain positive (0.058 e for CO and 0.076*e* for HCHO), indicating charge transfer from the gas molecules to the substrate. To quantitatively visualize the strain-modulated electronic and thermodynamic trends, we plot the bandgap and adsorption energy as functions of biaxial strain in Figure 8.

**Figure 8:**
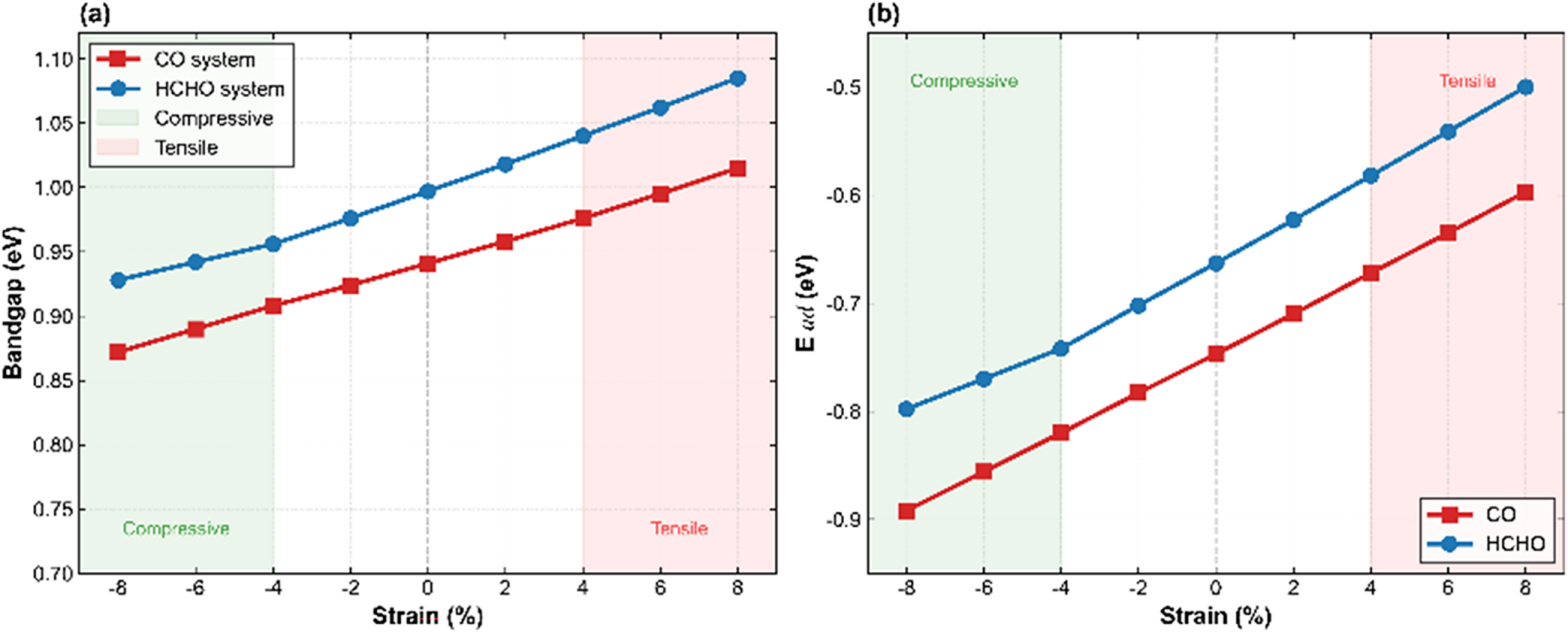
(a) Bandgap and (b) adsorption energy of the Pd-HfSSe monolayer as functions of biaxial strain. The shaded regions indicate compressive (green) and tensile (red) strain regimes.

As shown in Figure 8(a), the bandgap increases monotonically with strain for both adsorption systems. More importantly, Figure 8(b) reveals that the adsorption energy becomes more negative under compressive strain (−0.892 eV for CO at −8%) and less negative under tensile strain (−0.597 eV for CO at +8%), following the same trend as the charge transfer analyzed in Table 2. This confirms that compressive strain enhances the adsorption strength by shortening the Pd–gas bond length and promoting stronger orbital hybridization, while tensile strain weakens the adsorption through bond elongation and reduced hybridization. The consistency between *E*_ad_, *Q*_T_, and *E*_g_ variations establishes a coherent physical picture: strain modulates the gas-sensing performance primarily by altering the Pd–gas interaction geometry and the resulting electronic hybridization strength. The strain-dependent *E*_g_ and *Q*_T_ for both adsorption systems are presented in Figure 9.

**Figure 9:**
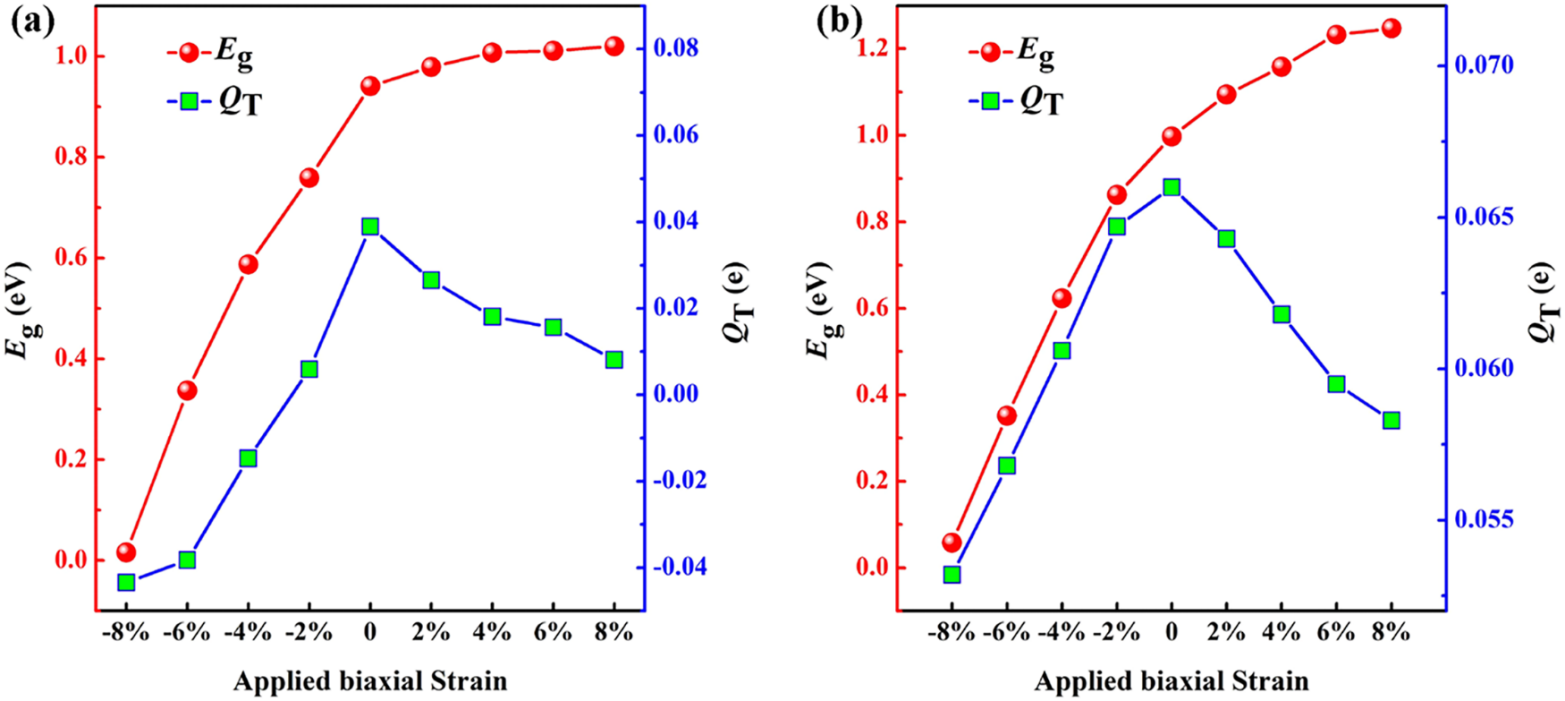
*E*_g_ and *Q*_T_ of (a) CO system and (b) HCHO system.

From Figure 9, it can be seen that compressive strains have a more obvious impact on the bandgap (*E*_g_) and adsorption parameter (*Q*_T_) than tensile strains. In other words, the changing trend of *E*_g_ and *Q*_T_ are much larger under compressive strains than under tensile strains. Furthermore, regardless of whether the strain is compressive or tensile, the electron-releasing character of the CO and HCHO molecules is weakened, leading to a reduction in *Q*_T_. Specifically, for the CO system, when the compressive strain exceeds −2%, charge transfer occurs from CO to the Pd-HfSSe monolayer, indicating charge-accepting behavior of the CO molecule under these conditions.

Moreover, compressive strains decrease the *E*_g_ of the Pd-HfSSe monolayer, while tensile strains increase it, and the rate of change under compressive strains is much larger than under tensile strains. That is, tensile strains reduce the sensing response of the Pd-HfSSe monolayer toward gas species, whereas compressive strains enhance it. Therefore, the Pd-HfSSe monolayer is a promising strain-tunable gas sensor for CO or HCHO detection under compressive strains, where the electrical response can be significantly enhanced. These findings suggest the strong potential of the Pd-HfSSe monolayer as a strain-tunable gas sensor for CO or HCHO detection under compressive strains with high sensitivity.^56^

## 4 Conclusions

This work performed first-principles simulations on the intrinsic HfSSe monolayer and its Pd-doped counterpart for CO and HCHO molecules to evaluate their potential as chemical gas sensors, with particular emphasis on CO as the primary detection target gas. The main findings are as follows:

(i) The *E*_ad2_ for the Pd-HfSSe/CO and Pd-HfSSe/HCHO systems is about 5.3 and 2.9 times higher than those of the pristine systems, highlighting the beneficial role of the Pd dopant in gas adsorption;
(ii) The BS analysis verifies the potential of Pd-HfSSe monolayer as a CO gas sensor with high sensitivity and good selectivity, while the HCHO response is relatively modest and should be interpreted with caution. The desirable recovery time (τCO = 4.09 s at 298 K) suggests its reusability for CO detection;
(iii) The Pd-HfSSe monolayer exhibits outstanding strain-modulated behavior, as reflected in the variations of *E*_g_ and *Q*_T_ upon gas adsorption, suggesting its potential as a tunable gas sensor.

This work provides a novel theoretical screening of HfSSe-based Janus materials for gas sensing applications, which also paves the way for further experimental investigations in many other fields.
